# Hepcidin Downregulation Correlates With Disease Aggressiveness And Immune Infiltration in Liver Cancers

**DOI:** 10.3389/fonc.2021.714756

**Published:** 2021-06-30

**Authors:** Jinhu Wang, Wang Liu, Jean C. Li, Mingyi Li, Benyi Li, Runzhi Zhu

**Affiliations:** ^1^ Department of Surgical Oncology, The Children’s Hospital, Zhejiang University School of Medicine, National Clinical Research Center for Child Health, Hangzhou, China; ^2^ Pediatric Oncology Program, Cancer Center, Zhejiang University, Hangzhou, China; ^3^ Department of Urology, The University of Kansas Medical Center, Kansas City, KS, United States; ^4^ Department of General Surgery, The Affiliated Hospital, Guangdong Medical University, Zhanjiang, China

**Keywords:** HAMP, liver cancer, cancer survival, disease progression, tumor immune infiltration

## Abstract

**Background:**

Hepcidin is a polypeptide hormone mainly produced by hepatocytes to modulate systemic iron balance. A drastic downregulation of the hepcidin gene was found in liver cancers. However, there is a paucity of information about the clinical significance of hepcidin gene downregulation in liver cancers.

**Methods:**

Hepcidin expression profiles were assessed using multiple public datasets *via* several bioinformatics platforms. Clinical and pathological information was utilized to stratify patients for comparison. Patient survival outcomes were evaluated using the Kaplan-Meier plotter, a meta-analysis tool. Tumor immune infiltration was analyzed using the single sample gene set enrichment analysis (ssGSEA) approach on the Cancer Genome Atlas (TCGA) dataset. Hepcidin antagonist Fursultiamine was used to treat liver cancer HepG2 and Huh7 cells together with Sorafenib.

**Results:**

Hepcidin gene was predominantly expressed in benign liver tissues but drastically decreased in liver cancer tissues. Hepcidin reduction in liver cancers correlated with risk factors like non-alcoholic fatty liver disease (NAFLD) and liver fibrosis, as well as cancer grade and tumor stage. Hepcidin downregulation was associated with a rapid cancer progression and worse disease-specific survival, especially in patients of the White race without alcohol consumption history. Hepcidin expression in liver cancer tissues positively correlated with the bone morphogenetic protein-6 (BPM6)/interleukin-6 (IL6) cytokines and cytotoxic immune infiltration. Blocking hepcidin action with its antagonist Fursultiamine moderately reduced Sorafenib-induced apoptotic cell death in HepG2 and Huh7 cells.

**Conclusion:**

Hepcidin downregulation in liver cancers correlated with liver cancer risk factors, cancer aggressiveness, cytotoxic immune cell infiltration, and patient survival outcomes. BMP6/IL6 pathway insufficiency is a potential cause of hepcidin downregulation in liver cancers.

## Introduction

Hepcidin (encoded by the hepcidin antimicrobial peptide gene, *HAMP*) is a polypeptide hormone with an important role in the regulation of systemic iron balance (1, 2). Hepcidin peptide was originally discovered in human urine and serum samples as an anti-microbial peptide based on its structural similarity (3, 4). Hepcidin was first named as a liver-expressed antimicrobial peptide (LEAP-1) due to the liver as the predominant site of production (3). In addition to the liver, other organs also produce hepcidin peptides to a very less extent, including the heart, brain, lung, prostate, adrenal, thyroid, urinary bladder, intestines, and kidney (3).

The human *HAMP* gene consists of three exons on chromosome 19q13.12 and encodes an 84 amino acid prepropeptide, which is then processed by the Furin convertase to yield the active hormone of hepcidin-25 peptide (5, 6). In general, hepcidin is produced at a relatively high rate (about 10 mg per day) but it is rapidly cleared through the kidney filtration from the circulation, resulting in an estimated half-life of only a few minutes (7). Excessive production of hepcidin peptide leads to iron-restricted anemia conditions while its deficiency is associated with iron overload diseases such as hereditary hemochromatosis and β-thalassemia (7). Hepcidin gene expression at the transcriptional level is regulated by several cellular signaling pathways, of which BMP6 and IL6 are the major stimulators (7, 8).

Studies in recent years reported that hepcidin was aberrantly expressed in multiple human cancers, including prostate, breast, lung, colon, brain, and liver cancers (9, 10). Especially, liver cancers showed a drastic reduction of hepcidin expression compared to benign liver tissues (11, 12), while all other cancers exhibited an increased level of hepcidin expression. Interestingly, hepcidin expression in tumor tissues at the mRNA levels was not correlated with the serum hepcidin levels in liver cancer patients (12). Hepcidin downregulation was linked to a higher rate of metastasis and worse disease-free status in hepatocellular carcinoma (HCC) patients (13). HAMP gene knockdown led to enhanced cancer cell proliferation and migration *in vitro* and xenograft tumor growth *in vivo* in liver cancer models (13). Consistently, treatment with exogenous hepcidin (Tilapia hepcidin TH2-3) potently inhibited fibrosarcoma cell growth (14). However, it is not fully clear whether hepcidin downregulation is related to liver cancer risk factors, tumor infiltration of immune cells, and patient disease-specific survival.

In this study, we took a comprehensive approach in analyzing hepcidin expression profiles in liver cancers using multiple gene expression databases available on publicly assessable bioinformatics platforms. Our analysis not only confirmed the hepcidin downregulation in malignant liver tissues compared to benign tissues, but also found a strong association between hepcidin downregulation and liver cancer risk factors, cancer grade, tumor stage, and patient survival. In addition, hepcidin expression strongly correlated with BMP6 and IL6 genes, as well as anti-cancer immune cell populations in the tumor microenvironment. Finally, blocking hepcidin action with an antagonist Fursultiamine reduced Sorafenib-induced cytotoxic effect in liver cancer cells.

## Materials and Methods

### Cell Lines, Culture Condition, and Experimental Reagents

Hepatocellular carcinoma cell lines HepG2 and Huh7 were purchased from the American Type Culture Collection (ATCC, Manassas, VA) and Health Science Research Resources Bank (JCRB0403, Osaka, Japan), respectively. These cells were kept in Dulbecco’s Modified Eagle Medium (DMEM) with 10% fetal bovine serum and 1% penicillin and streptomycin at 37°C in a 5% CO_2_ setting. Small chemicals Sorafenib (catalog #10009644) and Fursultiamine (catalog #33456) were purchased from Cayman Chemicals (Ann Arbor, MI). Antibodies to poly (ADP-ribose) polymerase (PARP, catalog #9532), Caspase-3 (catalog #9665), and Actin (catalog #58169) were purchased from Cell Signal Tech (Danvers, MA). A mouse monoclonal antibody to Ferroportin (FPN1) was obtained from ABCAM (catalog #239511). Horseradish peroxidase (HRP)-conjugated secondary antibodies and chemiluminescent reagents were purchased from Santa Cruz Biotech (Dallas, TX). All chemicals were initially dissolved in dimethyl sulfoxide (DMSO) as a stock solution and then diluted with cell culture media into the final concentration at a 1000-fold dilution. The chemical treatment period and the final concentrations were indicated in the figure legends.

### Cell Death Assessment and Western Blot Assays

The cell death rate was assessed using trypan blue exclusion assay, as described previously (15, 16). Briefly, cells were seeded at 5 x 10^4^ cells/well in a 6-well plate overnight and then treated with various reagents as indicated in the figure legend. After treatment, all cells were collected by trypsinization and stained with 0.4% trypan blue solution. Cells stained as blue were counted as dead cells and unstained cells were considered living cells.

To verify cell death by apoptosis, two classical apoptosis markers, PARP cleavage and Caspase-3 processing, were assessed in western blot assays (15). After treatment, cells were harvested in a cold phosphate-buffered saline (PBS) solution, and protein lysates were extracted using radioimmunoprecipitation assay (RIPA) buffer, as described (17). Equal amounts of proteins from each treatment were subjected to western blot assay with the antibodies as indicated in the figure.

### Assessment of Hepcidin Gene Expression From Public Datasets on Bioinformatic Platforms

To assess gene expression profiles in benign and malignant liver tissues, we used multiple bioinformatics platforms that harbor a variety of gene expression datasets. Oncomine™ platform (www.oncomine.org) has numerous cDNA microarray datasets and was used for the comparison between cancer and its normal counterpart tissues to generate a fold-change ratio (18). The Gene Expression Profiling Interactive Analysis (GEPIA) platform (gepia.cancer-pku.cn) was used for the pan-cancer analysis of gene expression profiles based on RNA sequencing datasets from the TCGA project (19). The XIANTAO platform (www.xiantao.love) was used to conduct a paired comparison of hepcidin expression in 50 liver cancers with case-matched adjacent benign tissues from the TCGA project.

### Correlation of Hepcidin Expression With Risk Factors and Clinicopathological Parameters

The cBioportal platform (www.cbioportal.org) was used to evaluate the correlation of hepcidin expression with liver cancer risk factors and clinicopathological parameters (20). This platform harbors 5 datasets of whole-genome RNA sequencing data including the TCGA project from 1070 liver cancer cases (21–24). Gene expression data were downloaded for multiple comparisons among different groups based on cancer risk factors and clinicopathological parameters.

### Survival Analysis in Patients WithHepcidin Downregulation

The Kaplan-Meier Plotter platform (www.kmplot.com) was used to analyze patient survival profiles based on hepcidin expression levels (25). This platform was built to serve as a meta-analysis tool using the RNA sequencing datasets collected from the Gene Expression Omnibus (GEO), European Genome-Phenome Archive (EGA), and TCGA projects. Patients were stratified based on tumor grades, disease stages, ethnic race groups, sex, ages at diagnosis, and common risk factors. The cutoff value for hepcidin expression to split patients as high or low expression subgroups were determined using the minimum p-value approach as described previously (26). The hazard ratio and its log-rank p-value for each comparison were calculated as described (26).

### Gene Expression Correlation and Enrichment Analysis

We used two steps to analyze hepcidin-related genes. First, the correlations of hepcidin expression with all human genes were calculated on the XIANTAO platform (www.xiantao.love) based on the whole transcriptome gene expression datasets (424 HCC patients) from the TCGA project. The top 596 genes with the Spearman rho above 0.30 were used for gene enrichment analysis of Kyoto Encyclopedia of Genes and Genomes (KEGG) pathways on the KEGG Orthology-Based Annotation System (KOBAS) platform (kobas.cbi.pku.edu.cn) (27). Then, the iron regulation-related genes were analyzed on the cBioportal platform using multiple RNA sequencing datasets including the TAGC project, as described earlier.

### Tumor Immune Infiltration Analysis

The tumor immune infiltration profiles were assessed on the XIANTAO platform. A total of 24 immune markers were used for distinguishing different immunocytes (28). The Spearman correlations of immunocyte markers with hepcidin expression levels were calculated using the single-sample GSEA (ssGSEA) approach (29).

### HAMP Gene Profiling in GDS Datasets and DNA Methylation Analysis

Two GEO datasets, GDS2006 (30) and GDS1385 (31), were used to assess gene expression profiles in animal models. *HAMP/BMP6* gene expression data were downloaded from the NCBI/GEO site and were normalized with the *ACTB* gene expression level as an internal control.

Gene promoter methylation was analyzed using the University of Alabama Cancer Database (UALCAN) platform (ualcan.path.uab.edu) (32). The methylation score of the gene promoter DNA was calculated within the range from 0 (unmethylated) to 1 (fully methylated). The cut-off value of 0.5-0.7 was considered to indicate hypermethylation, while the value of 0.25-0.3 was considered hypomethylation (33, 34).

### Data Presentation and Statistical Analysis

All cell-based experiments were repeated three times. The quantitative data downloaded from bioinformatics platforms were presented as the mean plus the standard error of the mean (SEM). The images from the western blot assay were representative of multiple blots. The graphic images were generated on the bioinformatics platforms. Statistical analysis for multiple group data was conducted using the Analysis of Variance (ANOVA) method followed by a Student *t*-test or Wilcoxon rank-sum test for the comparison between two groups. Statistical calculation was conducted using the GraphPad Prism (version 9.1.0, San Diego, CA), and the p < 0.05 was considered statistically significant.

## Results

### Hepcidin Expression Is Significantly Reduced in Human Liver Cancers

**Figure 1:**
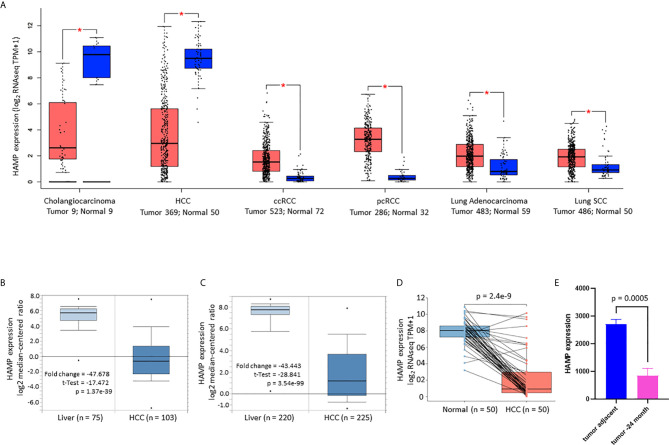
Hepcidin expression is drastically reduced in liver cancers. **(A)** Hepcidin expression profiles in human cancers were compared with normal tissues using the TCGA dataset on the GEPIA platform (19). Case numbers in each group were listed at the bottom of the figure. The asterisks indicate a statistically significant difference (p < 0.05, Wilcoxon rank-sum test). HCC, hepatocellular carcinoma; ccRCC, clear cell renal cell carcinoma; pcRCC, papillary cell renal cell carcinoma; Lung SCC, lung squamous cell carcinoma. **(B, C)** Hepcidin expression in liver cancers was assessed in two cDNA microarray datasets (37, 38). The comparison between normal and malignant liver tissues was conducted using a Student *t*-test. **(D)** Hepcidin expression in human liver cancers was compared with matched tumor-adjacent benign tissues in 50 cases from the TCGA project. The p-value was from a paired *t*-test. Note: only three cases showed a slight increase in cancer tissue compared to the benign compartment. **(E)** Hepcidin expression data was derived from the GDS2006 dataset in the *Tnixp*-deficient mice with spontaneous liver cancer (30). Hepcidin expression levels were normalized with *ACTB* gene levels as an internal control (n = 3).

A comprehensive analysis of hepcidin expression profiles was conducted using publicly assessable datasets from multiple bioinformatics platforms. Our analyses on two different datasets (35, 36) discovered that hepcidin expression at the mRNA level was predominantly high in the liver, followed by the brain, pancreas, parotid gland, heart, and adrenal gland (**Supplemental Figure S1**). In liver cancers, hepcidin expression was drastically reduced whereas kidney and lung cancers showed a significant increase (**Figure 1A**). These results of hepcidin downregulation in liver cancers were verified using another two datasets derived from cDNA microarray assay (37, 38), which showed hepcidin downregulation about 43-47 folds in HCC tissues compared to benign liver tissues (**Figures 1B, C**), similar to previous reports (11, 12). Hepcidin downregulation was also confirmed in a pairwise comparison of 50 liver cancer tissues with matched adjacent benign tissues derived from the TACG project (**Figure 1D**). Consistently, hepcidin downregulation was also observed in an experimental liver cancer model of the thioredoxin interacting protein (*Tnixp*)-deficient mice (30) (**Figure 1E**). Altogether, our analyses demonstrated that hepcidin gene expression was severely attenuated in liver cancers including hepatocellular carcinoma and bile duct cancers.

### Hepcidin Downregulation Correlates With Risk Factors and Aggressiveness in Liver Cancers

**Figure 2:**
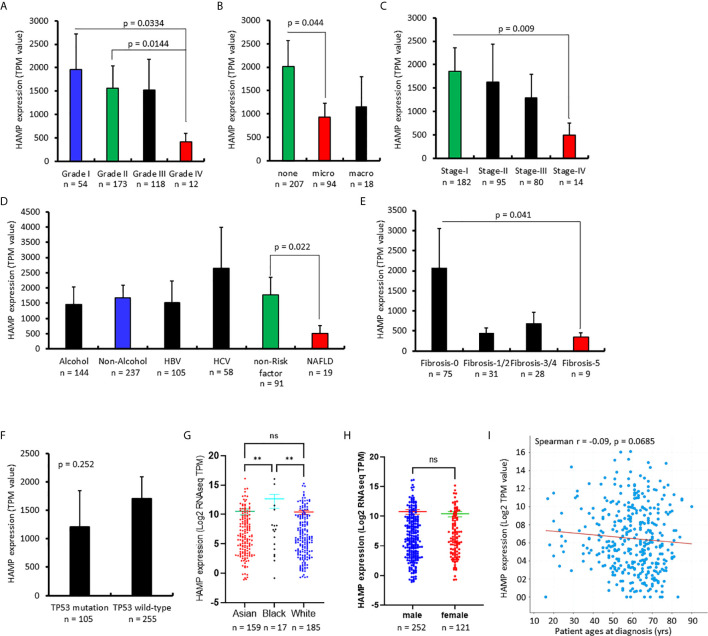
Hepcidin downregulation correlates with cancer risk factors and aggressiveness in liver cancers. **(A–H)**. Hepcidin expression (TPM value) from four datasets (21–24) plus the TCGA project was downloaded from the cBioportal platform. Case numbers in each group were listed at the bottom of the figure. The p-value as shown was from the Student *t*-test. The double asterisks in **(G)** indicate a significant difference (p < 0.01). ns, no significance. **(I)** Correlation of hepcidin expression levels with patient ages was analyzed on the cBioportal platform with 1070 cases of HCC patients.

We then focused our investigation on hepatocellular carcinomas (HCC) and explored the clinical significance of hepcidin expression related to patient survival and disease progression. Based on the RNA sequencing data, hepcidin expression was significantly reduced in Grade-IV compared to Grade-I/II HCCs (**Figure 2A**), in microvascular-invaded tumors compared to non-invaded tumors (**Figure 2B**), in Stage-IV compared to Stage-I cases (**Figure 2C**), in patients with non-alcohol fatty liver disease (NAFLD) compared to patients without any risk factors (**Figure 2D**), as well as in patients with severe liver fibrosis compared to non-fibrosis patients (**Figure 2E**). Despite a previous report showed a *TP53*-dependent transcriptional regulation of hepcidin gene expression in human liver cancer cells (39), there was no significant difference in hepcidin expression between *TP53* gene mutation and wild-type HCC cases (**Figure 2F**). In addition, hepcidin expression was significantly higher in the Black race group compared to the White and Asian race groups (**Figure 2G**). However, there was no significant difference in hepcidin expression between sex (**Figure 2H**) and no significant correlation between hepcidin gene expression and patient ages at diagnosis (**Figure 2I**).

### Hepcidin Downregulation Correlates With a Worse Survival Outcome in Liver Cancer Patients

**Figure 3:**
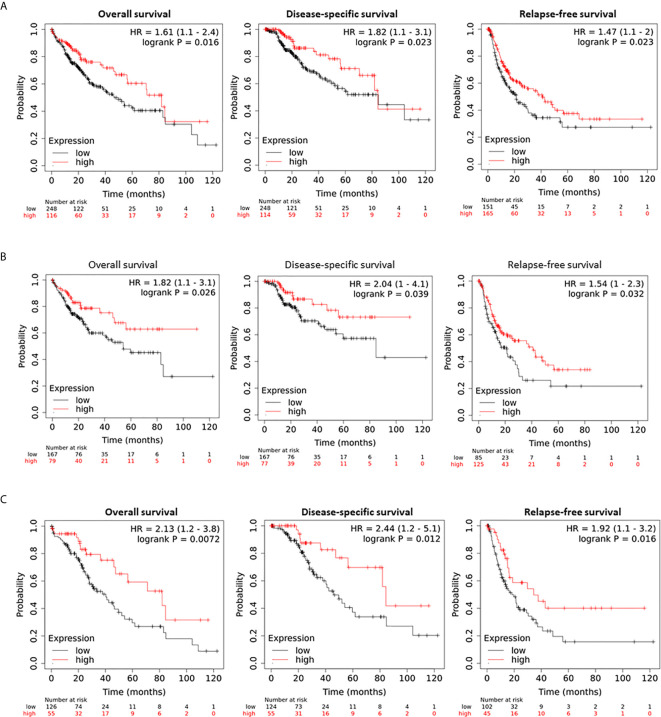
Hepcidin correlates with a worse survival outcome in liver cancer patients. **(A)** Patient survival outcome was analyzed on the Kaplan-Meier Plotter platform. Hepcidin expression levels were ranged as 0-120133 and the cutoff value for hepcidin expression was set as 501 in the overall and disease-specific survival analysis and 82 for the relapse-free survival using the minimum p-value approach as described (26). Case numbers in each group were listed at the bottom of the figure. **(B)** The cutoff value for hepcidin expression was 48 in the relapse-free survival comparison. **(C)** Hepcidin expression range was 0-66150 and the cutoff value was 511 for all three comparisons.

The effect of hepcidin downregulation on patient survival was examined using the Kaplan-Meier Plotter platform (26). As shown in **Figure 3A**, in a cohort of 364 HCC cases, patients with lower hepcidin expression (HAMP^low^ group) showed a significantly worse overall, disease-specific, and relapse-free survival. The average hazard ratio (HR) was 1.61, 1.82, and 1.47, respectively. These effects were exhibited only in male patients with an increased HR value of 1.82, 2.04, and 1.54, respectively, for overall, disease-specific and relapse-free survival (**Figure 3B**). Further analysis revealed that the ethnic race of the White patients showed a strong effect of hepcidin downregulation (HAMP^low^ group) on patient survival, including overall (HR = 2.13), disease-specific (HR = 2.44), and relapse-free (HR = 1.92) survival (**Figure 3C**).

**Figure 4:**
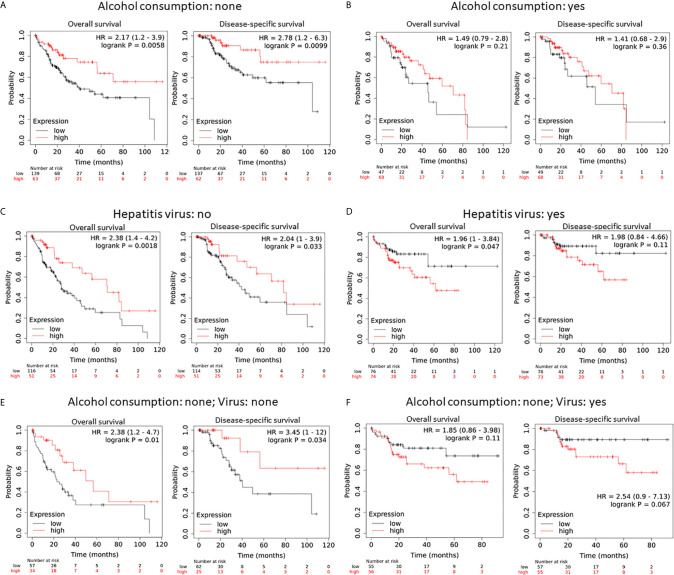
Hepcidin downregulation is a worse survival factor in non-alcoholic liver cancer patients without viral hepatitis. **(A, B)** The survival status was analyzed on the Kaplan-Meier Plotter platform in patients with or without alcohol consumption. In panel A, the hepcidin expression range (RNA sequencing data) was 0-116709 and the cutoff value was 542. In panel B, the hepcidin expression range was 0-120133 and the cutoff value was 68 using the minimum p-value approach, as described (26). Case numbers in each group were listed at the bottom of the figure. **(C, D)** The survival status was analyzed in patients with or without viral hepatitis. The cutoff value for hepcidin expression was set as 440 (range 0-58791) in panel C and 168 in panel D (range 0-120133). **(E, F)** The survival status was analyzed in none-alcoholic patients with or without viral hepatitis. The cutoff value for hepcidin expression was 140 (range 0-49344) for the overall survival but 542 for the disease-free survival in **(E)**. The cutoff value in **D** was 168 (range 0-116709).

Since alcohol consumption and chronic viral infection are common risk factors for liver cancers (40), we analyzed if hepcidin downregulation had any additional effect on the patient’s survival. Hepcidin downregulation (HAMP^low^ group) had a significantly worsening effect on overall and disease-specific survival in patients without alcohol consumption. The HR value was 2.17 and 2.78, respectively (**Figure 4A**). However, these worsening effects were not significant in patients with alcohol consumption (**Figure 4B**). Meanwhile, patients without viral hepatitis also rendered a worse overall and disease-free survival status in the hepcidin downregulation (HAMP^low^) group (**Figure 4C**). Conversely, patients with viral hepatitis showed a favorite overall (but not disease-specific) survival in the hepcidin downregulation (HAMP^low^) group (**Figure 4D**). When combining alcohol consumption and viral hepatitis, hepcidin downregulation had a significantly worsening effect on overall (HR = 2.38) and disease-specific (HR = 3.45) survival in patients without both alcohol consumption and hepatitis (**Figure 4E**). These effects were reverted in patients without alcohol consumption but with viral hepatitis, although it was not statistically significant (**Figure 4F**). These data suggest that hepcidin downregulation is a worse prognosis factor in HCC patients without alcohol consumption and that viral infection reverted it to a favorite survival factor. The mechanism underlying these events is under further investigation.

### Hepcidin Expression Correlates With Iron Modulating Factor BMP6/IL6 in Liver Cancers

To explore hepcidin-associated genes, we conducted a correlation analysis based on the TCGA dataset. A total of 596 genes were identified as positively associated genes and 110 genes as negatively associated genes with a Spearman rho value above +3.0 or below -3.0, q < 0.001, **Supplemental Table S1**). Gene expression enrichment analysis revealed that the most significantly enriched clusters were inflammatory cytokines/chemokines, TGFβ pathway, and immune system (**Supplementary Tables S2**, **S3** and **Figure S2**). Among those positively associated genes, BMP family genes (BMP5/6/BMPER) and inflammatory cytokines (IL6/33), along with their receptors BPMR1B/ALK6, ACVRL1/ALK1, and IL1RL1, showed a strong positive correlation with HAMP expression (**Table 1**). These data are supported by previous reports that BMP6 and IL6 pathways regulate hepcidin expression at the transcription level in hepatocytes (7, 41, 42).

**Table 1:** Correlations of HAMP with iron regulation-related genes.

Iron-regulating factors	cBioportal platform Spearman r (p value)	XIANTAO platform Spearman r (p value)
**BMP5**	0.34 (8.81e-12)	0.38 (2.40e-14)
**BMP6**	0.42 (5.76e-17)	0.31(1.03e-9)
**BMPER**	0.42 (1.57e-17)	0.39 (8.14e-15)
**ACVRLl/ALKl**	0.43 (7.63e-18)	0.32 (1.99e-10)
**BMPR1B/ALK6**	0.35 (1.67e-12)	0.34 (8.72e-12)
**IL6**	0.34 (8.49e-12)	0.33 (7.47e-11)
**IL33**	0.37 (2.53e-13)	0.31(5.86e-10)
**lllRLl**	0.31(1.47e-9)	0.31(4.21e-10)
TMPRSS6/MT2	0.1(0.0583)	0.013 (0.801)
TFRC	-0.16 (2.489e-3)	-0.148 (0.004)
HFE/HFE1	-0.14 (7.681e-3)	-0.148 (0.004)
HJV/HFE2	0.06 (0.214)	-0.037 (0.472)
TFR2/HFE3	0.21(5.196e-5)	0.084 (0.105)
SLC40A1/H FE4/FPN1	-0.09 (0.0764)	-0.134 (0.009)
SLC11A2	-0.23 (6.736e-6)	-0.216 (2.49e-5)
BMPR1NALK3	-0.20 (8.972e-5)	-0.189 (2.34e-4)
BMPR2	0.00 (0.924)	-0.039 (0.44)
ACVR1/ALK2	-0.09 (0.0736)	-0.107 (0.039)
ACVR1B	-0.03 (0.503)	-0.083 (0.107)
ACVR1C	-0.04 (0.494)	-0.107 (0.039)
ACVR2B/ACTRIIB	-0.05 (0.310)	-0.107 (0.039)
SMAD7	0.21(5.086e-5)	0.146 (4.55e-3)
NE01	-0.12 (0.0178)	-0.124 (0.016)

**Figure 5:**
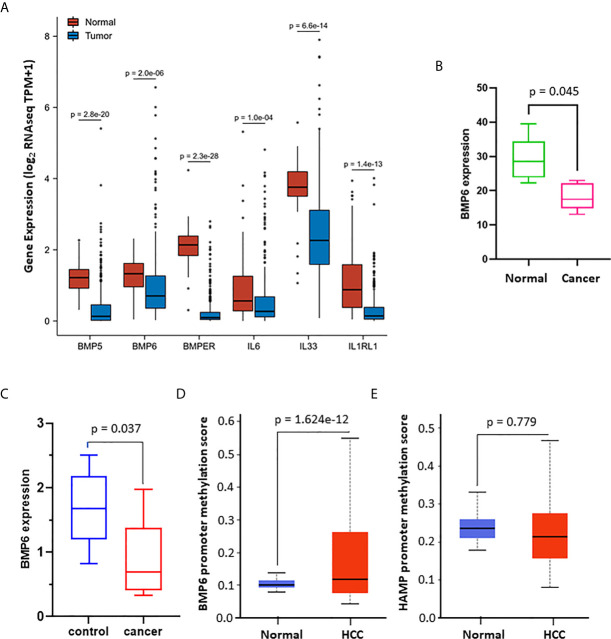
Hepcidin expression is positively associated with iron regulation-related genes. **(A)** Gene expression profiles were analyzed using the TCGA dataset on the XIANTAO platform (424 liver cancer cases, 50 benign liver tissues). The p-value was from a Wilcoxon rank-sum test. **(B)** BMP6 gene expression data were downloaded from GEO dataset GDS2006, which was generated from the thioredoxin interacting protein-deficient mice with spontaneous liver cancer (30). BMP6 expression levels were normalized with the *ACTB* gene levels as an internal control (n = 3). The p-value was from a Student *t*-test. **(C)** BMP6 gene expression data were downloaded from the GEO dataset GDS1385, which was generated from a rat liver cancer model induced by long-term feeding of choline-deficient/L-amino acid-defined diet (31). BMP6 expression levels were normalized with the *ACTB* gene levels as an internal control (n = 3). The p-value was from a Student *t*-test. **(D, E)** Promoter DNA methylation was analyzed using the TCGA dataset (n = 424) on the UALCAN platform (32). The p-value was from a Student *t*-test.

To determine the significance of these strong correlations, we assessed if their expression was also altered in human liver cancer tissues. Our analysis revealed that these five cytokines plus IL1RL1 genes were significantly downregulated in HCC tissues compared to normal liver tissues (**Figure 5A**), along with the HAMP gene (**Figure 1**). To further elucidate the significance of BMP6 downregulation in liver cancers, we analyzed BMP6 expression levels in experimental liver cancer models due to *Tnixp* gene deficiency (30). As shown in **Figure 5B**, BMP6 expression was significantly reduced in liver cancers of *Tnixp*-mutant mice, which is in parallel with hepcidin reduction (**Figure 1E**). In another model of rat liver cancer after long-term feeding of choline-deficient/L-amino acid-defined diet (31), BMP6 expression was also significantly reduced in liver cancers compared to normal liver tissues (**Figure 5C**). These data suggest that BMP6 expression was reduced during liver cancer development in animal models, possibly contributing to hepcidin downregulation.

To explore a potential mechanism of BMP6 downregulation, we analyzed gene promoter methylation of HAMP and BMP6 using the TCGA dataset. The results showed that BMP6 but not HAMP gene promoter was highly methylated in human HCC tissues compared to normal liver tissues (**Figures 5D, E**). These data suggest that DNA hypermethylation on BMP6 gene promotor is potentially involved in BMP6 downregulation in HCC tissues.

### Hepcidin Expression Correlates With Cytotoxic Immune Cell Infiltration in HCC Tissues

**Figure 6:**
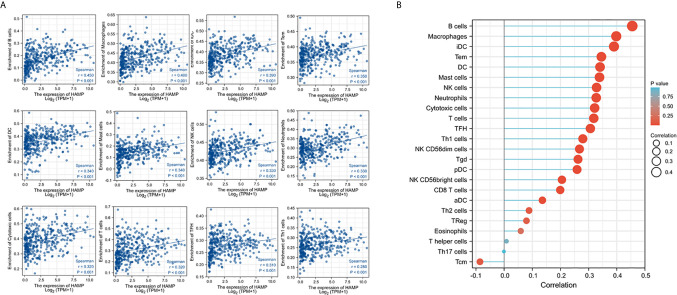
Hepcidin expression positively correlates with anti-cancer immune infiltration. The correlation of hepcidin expression levels with the immune infiltration in liver cancer tissues was analyzed using the TCGA dataset on the XIANTAO platform (n = 424). Strong correlations (Spearman rho > 0.3) were shown from 11 immune types in **(A)**. **(B)** showed the correlations for all 24 immune cell types. iDC, immature DC; Tem, T effector memory; TFH, T follicular helper; Tgd, T gamma delta; pDC, plasmacytoid DC; aDC, activated DC; Tcm, T central memory.

Immune infiltration in the tumor microenvironment is a key factor in determining anti-cancer efficacy and patient outcome (43). We assessed the correlation of hepcidin expression with immune infiltration profiles in HCC tissues. The results showed that among 24 types of infiltrating immune cells, 11 types showed a strong positive correlation with hepcidin expression (Spearman rho > 3.0, p < 0.05, **Figure 6A**). All these positively correlated types of immune cells possess anti-cancer properties (44), including cytotoxic cells, T cells, B cells, NK cells, Neutrophils, and DC cells (**Figure 6B**). These data indicate that hepcidin downregulation might be accompanied by a reduced anti-cancer immune infiltration, resulting in a worse survival outcome.

### Hepcidin Antagonist Fursultiamine Reduces Sorafenib-Induced Cell Death in HCC Cells

**Figure 7:**
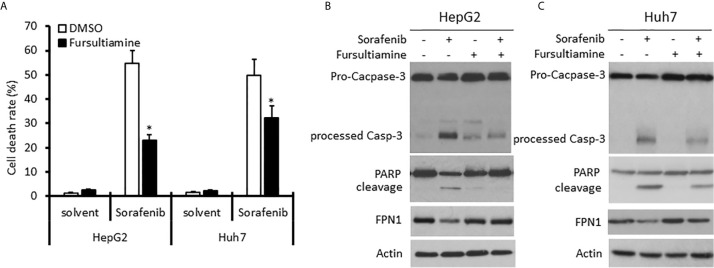
Hepcidin antagonist pretreatment reduces Sorafenib-induced cell death in HCC cells. HepG2 and Huh7 cells were pre-treated with Fursultiamine (10 µM) for 30 minutes, followed by Sorafenib (5 µM) treatment for 24 h. Cells were harvested for trypan blue exclusion assay **(A)** or western blot assays with the antibodies as indicated **(B, C)**. Actin blots served as the protein loading control. The asterisk indicates a significant difference compared to the DMSO control (p < 0.05, Student *t*-test).

Sorafenib has been shown to enhance hepcidin expression in HCC cells (45). We then reasoned if blocking hepcidin action interferes with Sorafenib-induced cell death. Hepcidin antagonist Fursultiamine (46) was used to pre-treat HepG2 and Huh7 cells for 30 min, followed by Sorafenib treatment for 24 h. As shown in **Figure 7A**, Sorafenib treatment induced a strong cell death-inducing effect on both HCC cell lines. Fursultiamine pretreatment moderately ameliorated Sorafenib-induced cell death. In parallel, Sorafenib-induced apoptosis, as assessed by Caspase-3 processing and PARP cleavage, was largely reduced in both cell types pre-treated with Fursultiamine (**Figures 7B, C**). This result was supported by a previous report that Sorafenib-induced cell death is an apoptotic response (47). In addition, Sorafenib treatment also moderately reduced Ferroportin (FPN1, *SLC40A1*) protein levels, which was largely reversed in Fursultiamine pre-treated cells. These data indicate that hepcidin induction by Sorafenib treatment might be involved in Sorafenib’s anti-cancer effect.

## Discussion

In this study, we analyzed hepcidin expression in liver cancers. Our results revealed that hepcidin was predominantly expressed in normal liver tissues but drastically decreased in liver cancers. Hepcidin reduction in liver cancers correlated with cancer grade and stage, as well as risk factors of NAFLD and fibrosis. Importantly, hepcidin downregulation was associated with worse survival in liver cancer patients, especially in patients of the White race without alcohol consumption history. Furthermore, hepcidin expression was tightly associated with BMP6 proteins and IL6 cytokines, as well as anti-cancer immune infiltration in liver cancer tissues.

It was reported that hepcidin expression was reduced about 33-35 folds in HCC tissues compared to benign liver tissues (11, 12). A recent study showed that hepcidin expression was reduced in metastatic HCC tumors and that the relapse-free survival was slightly different between HAMP^high^ or HAMP^low^ patients (13). In this study, our results showed a nice agreement of hepcidin downregulation (43-47 folds reduction) in HCC tissues. In addition, our results showed a significant reduction of hepcidin expression in high-grade and late-stage cancers, as well as a significantly worse survival outcome in HAMP^low^ patients. Interestingly, we also noticed for the first time that there was an ethnic disparity for the effect of hepcidin downregulation on patient survival between the White and other race groups. Furthermore, patients from the Black race showed a slightly but significantly higher level of hepcidin expression than the Asian or the White race. The underlying mechanism for this ethnic disparity is under further investigation by our team.

Currently, the mechanism for hepcidin downregulation in liver cancers is not clear. It was reported that the HAMP gene promoter was hypermethylated in HCC cell lines (48), but our analysis did not yield a significant increase in HAMP promoter methylation. Although previous reports showed that alcoholic hepatitis, viral infection, and TP53 affected hepcidin expression in liver cells (39, 49–51), our data did not show a significant correlation between these factors and hepcidin expression. Among the risk factors for human liver cancers, HCC patients with NAFLD or liver fibrosis showed a significant hepcidin downregulation. These data suggest that chronic hepatocyte injury is a potential factor for HAMP downregulation since hepcidin expression is associated with a healthy liver function (52).

BMP6 as the major regulator of iron homeostasis regulates hepcidin expression in liver cells through SMAD transcription factors and other proteins like TMPRSS6 or HJV (53–56). In this study, we identified five genes in the BMP pathway, BMP5, BMP6, BMPER, BMPR1B, and ACVRL1, that had a strong and significant correlation with hepcidin expression. On the other hand, BMP6 expression was significantly reduced in parallel with hepcidin downregulation in both human and animal HCC tissues from clinical specimens and experimental animal models. Currently, there is a paucity of reports about BMP protein expression in liver cancers. There was only one study showing a reduced BMP6 expression due to BMP6 promoter hypermethylation in human liver cancer tissues (57). In agreement with this report, we also found a 2.2-fold reduction of BMP6 expression in HCC tissues, possibly due to BMP6 gene promoter hypermethylation. Altogether, our results indicate that BMP6/HAMP insufficiency might be an important factor in liver cancer development or progression.

Immune infiltrating cells are the major components in the tumor microenvironment and innate immune cells including NK cells, mast cells, neutrophils, macrophages, and DC cells are critical in tumor suppression and surveillance (44). In addition, the adaptive immune cells including B cell, cytotoxic T cell, T helper cells, and T memory cells are immune effector cells, important for anti-cancer immunotherapy (58). In this study, our analysis discovered for the first time that hepcidin expression was positively associated with tumor infiltration of 11 immune effector cells from both innate and adaptive immune systems. In other words, hepcidin downregulation might be accompanied by reduced immune surveillance and even less responsive to immunotherapy in the liver cancer microenvironment. A further interactive investigation is warranted to understand the promoting effect of liver cancer cell-delivered hepcidin peptides on immune infiltration profiles.

Targeted therapy with multi-kinase inhibitors such as Sorafenib is the standard of care for advanced liver cancers in the last 15 years (59). Interestingly, Sorafenib was found to enhance hepcidin gene expression in liver cancer Huh7 cells, together with several other kinase inhibitors, including phosphoinositide 3-kinase (PI3K), mechanistic target of rapamycin (mTOR), Ras/mitogen-activated protein kinase (MAPK), and AMP-activated protein kinase (AMPK) pathway inhibitors (45). In this study, blocking HAMP action by its antagonist Fursultiamine (46) moderately ameliorated Sorafenib-induced cell death, indicating that Sorafenib-induced HAMP expression might be involved in its cytotoxic effect on liver cancer cells (47). Our results were supported by recent studies using the hepcidin shRNA approach (13) and an exogenous hepcidin peptide approach (14). It is worthy to determine the potential implication of HAMP expression as a biomarker for targeted therapy or immunotherapy in clinical settings.

## Conclusion

Our investigation revealed that hepcidin is predominantly expressed in the liver and its expression was altered in several human cancer types. In human liver cancers, hepcidin expression was drastically reduced, and hepcidin downregulation correlated with cancer grade and disease stage. Two liver cancer risk factors, NAFLD and fibrosis, were strongly associated with hepcidin downregulation. Hepcidin downregulation had a significantly worsening effect on patient survival outcome, especially in the White race group without alcohol consumption and viral history. Hepcidin gene expression correlated with BMP6 and IL6 expression, as well as tumor-infiltrating anti-cancer immune cell populations. Blocking hepcidin action reduced Sorafenib-induced cytotoxic effect in HCC cells. Our study indicates that BMP6/HAMP pathway insufficiency is associated with a worsen survival outcome in HCC patients and that hepcidin supplementation might improve patient outcome, but a further interventional study is needed to draw a clear conclusion.

## Data Availability Statement

The original contributions presented in the study are included in the article/**Supplementary Material**. Further inquiries can be directed to the corresponding authors.

## Author Contributions

RZ and BL designed the study. JW, RZ, ML, and BL analyzed the bioinformatics data. JW, WL, and JL performed cell-based experiments. BL and RZ performed the statistical analysis and generated figures and tables. JW, RZ, and BL drafted the manuscript. All authors contributed to the article and approved the submitted version.

## Funding

JL was a recipient of the K-INBRE Entrepreneurial Scholar Award in 2020.

## Conflict of Interest

The authors declare that the research was conducted in the absence of any commercial or financial relationships that could be construed as a potential conflict of interest.

## Supplementary Material

Supplementary Figure 1 Hepcidin gene is predominantly expressed in normal liver tissues. Hepcidin expression profiles were assessed with two databases (35, 60) on the Oncomine™ platform (18). These two datasets contained 123 (35) and 504 (60) samples, respectively. The cDNA microarray was conducted using the Human Genome U133 Plus 2.0 array of 19574 genes in the Roth dataset (60). There were 14430 measured genes in the Shyamsundar dataset (35).

Supplementary Figure 2 Hepcidin gene expression is positively associated with iron regulation-related genes. The correlation of hepcidin gene expression with the whole transcriptome was analyzed using the TCGA dataset on the XIANTAO platform. The top 596 correlated genes with hepcidin expression (Spearman rho > 3.0 with q value < 0.05) were used for gene enrichment analysis on the KOBAS platform. Data visualization for KEGG pathways was carried out based on the Enrich ratio (left panel) and -log_10_[p value] (right panel).
