# Supplementary figures and images for: Hepcidin Downregulation Correlates With Disease Aggressiveness And Immune Infiltration in Liver Cancers
Source: Front Oncol. 2021 Jun 30;11:714756. doi: 10.3389/fonc.2021.714756 (PMC8278784; doi:10.3389/fonc.2021.714756)

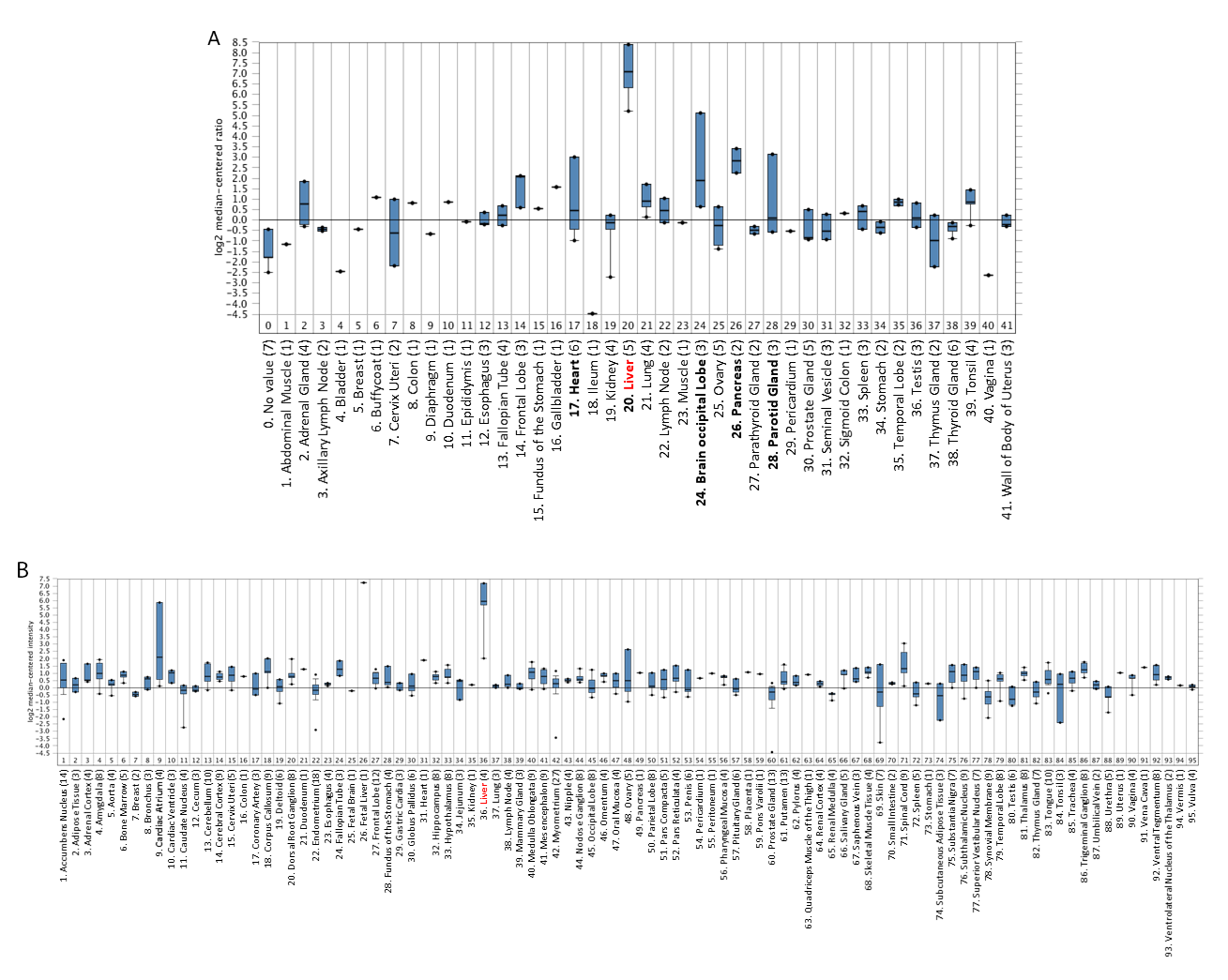

Supplement: Supplementary Figure 1 — Hepcidin gene is predominantly expressed in normal liver tissues. Hepcidin expression profiles were assessed with two databases (35, 60) on the Oncomine™ platform (18). These two datasets contained 123 (35) and 504 (60) samples, respectively. The cDNA microarray was conducted using the Human Genome U133 Plus 2.0 array of 19574 genes in the Roth dataset (60). There were 14430 measured genes in the Shyamsundar dataset (35). [file Image_1.tif]

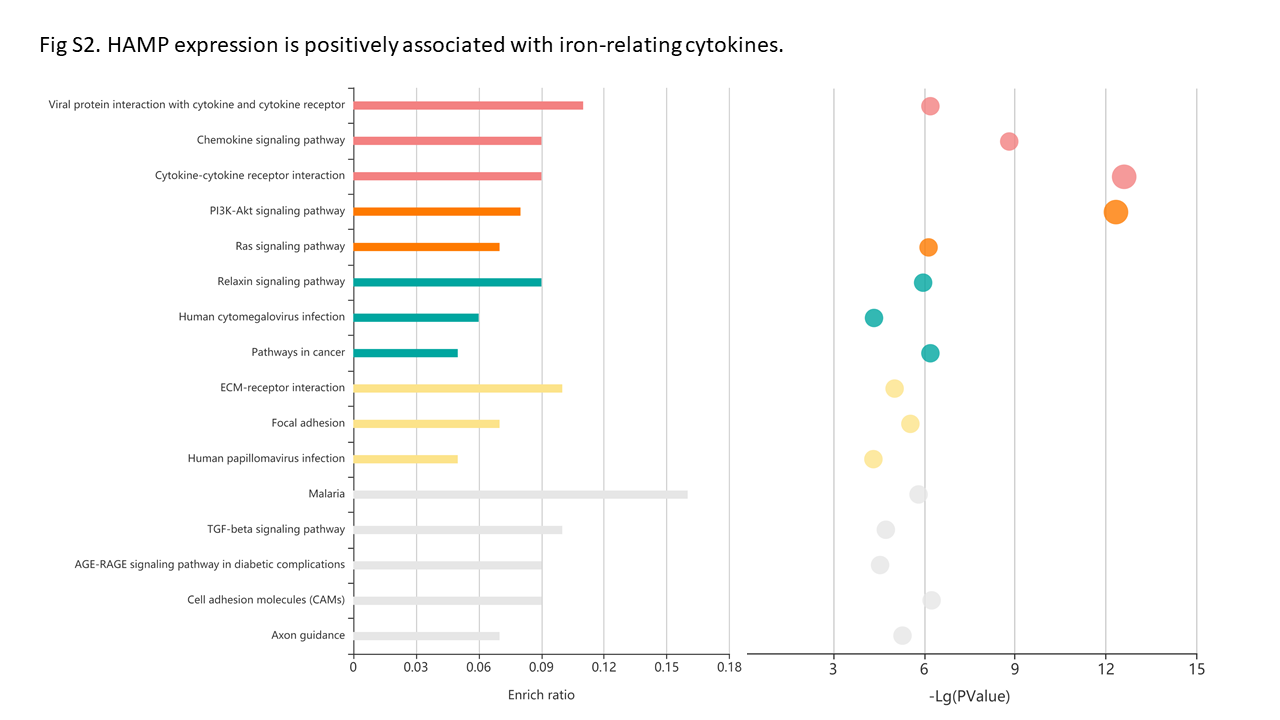

Supplement: Supplementary Figure 2 — Hepcidin gene expression is positively associated with iron regulation-related genes. The correlation of hepcidin gene expression with the whole transcriptome was analyzed using the TCGA dataset on the XIANTAO platform. The top 596 correlated genes with hepcidin expression (Spearman rho > 3.0 with q value < 0.05) were used for gene enrichment analysis on the KOBAS platform. Data visualization for KEGG pathways was carried out based on the Enrich ratio (left panel) and -log10[p value] (right panel). [file Image_2.tif]
